# Effects of treatment modalities for Graves’ hyperthyroidism on Graves’ orbitopathy: a 2015 Italian Society of Endocrinology Consensus Statement

**DOI:** 10.1007/s40618-015-0257-z

**Published:** 2015-02-27

**Authors:** L. Bartalena, P. E. Macchia, C. Marcocci, M. Salvi, F. Vermiglio

**Affiliations:** 1 Endocrine Unit, Department of Clinical and Experimental Medicine, University of Insubria, Ospedale di Circolo, Viale Borri, 57, 21100 Varese, Italy; 2 Department of Clinical Medicine and Surgery, University of Naples “Federico II”, Naples, Italy; 3 Department of Clinical and Experimental Medicine, University of Pisa, Pisa, Italy; 4 Department of Clinical Sciences and Community Health, Graves’ Orbitopathy Center, Fondazione Ca’ Granda IRCCS and University of Milan, Milan, Italy; 5 Department of Clinical and Experimental Medicine, University of Messina, Messina, Italy

**Keywords:** Graves’ disease, Graves’ orbitopathy, Antithyroid drugs, Thionamides, Radioiodine, Thyroidectomy, Glucocorticoids

## Introduction

Graves’ disease is the most frequent form of hyperthyroidism in iodine sufficient countries [1], and Graves’ orbitopathy (GO) is its most important and common extrathyroidal manifestation [2], affecting about 25 % of patients [3]. Although GO is generally mild and rarely progressive [4], thyroid dysfunction, both hyperthyroidism and hypothyroidism, can influence its course. GO has been reported to improve after correction of hyperthyroidism with antithyroid drug (ATD) treatment [5], or to occur or worsen after a period of uncontrolled hypothyroidism [6]. Accordingly, the European Group on Graves’ Orbitopathy (EUGOGO) Consensus Statement few years ago recommended that restoration and maintenance of euthyroidism are priorities in Graves’ disease patients with GO [7]. How to treat hyperthyroidism when GO is present is, however, a challenging dilemma [8]. Are current modalities for hyperthyroidism [ATDs, radioiodine (RAI), thyroidectomy] per se capable to affect the course of GO? If orbital disease is present, is it preferable to control hyperthyroidism with ATDs or may thyroid ablation (RAI, thyroidectomy, alone or in association) be advantageous by removing factors (thyroid autoreactive lymphocytes, thyroid antigens) that may promote the occurrence and/or progression of GO? To address these questions, the Italian Society of Endocrinology established a task force of experts with the aim of reviewing the available literature and drawing conclusions based on evidence summary of recommendations is presented in Table 1.

**Table 1:** Summary of recommendations

Recommendation number	Statement	Strength and level of evidence
1	In patients with newly diagnosed Graves’ hyperthyroidism, euthyroidism should be promptly restored by antithyroid drugs, and then stably maintained	1, ØØØØ
2	Thyroid status should be assessed frequently during the initial phase of antithyroid drug treatment and regularly thereafter, to avoid fluctuations in thyroid status potentially detrimental for GO	1, ØØØO
3	Steroid prophylaxis is recommended in patients receiving radioiodine treatment, if mild and active GO preexists or there are risk factors for radioiodine-associated GO development or progression	1, ØØØØ
4	Pros and cons of steroid prophylaxis after radioiodine treatment should be thoroughly discussed also with patients with absent or inactive GO prior to radioiodine treatment	1, ØOOO
5	If surgery is selected, near-total/thyroid thyroidectomy should be preferred to subtotal thyroidectomy, because the former is associated with a higher rate of successful treatment of hyperthyroidism, with no differences in the outcome of GO; steroid prophylaxis is not required	1, ØØØØ
6	If surgery for Graves’ hyperthyroidism is selected in patients with GO, post-operative remnant ablation may be considered, because this inactivates the disease earlier and allows prompter rehabilitative surgery, if needed	2, ØØØO
7	Patients who have mild and active GO and are treated with antithyroid drugs should receive a 6-month selenium supplementation	1, ØØØO
8	The modality of treatment for hyperthyroidism in patients with mild and active GO should be selected independently of GO	1, ØOOO
9	The modality of treatment for hyperthyroidism in patients with mild and inactive GO should be selected independently of GO	1, ØOOO
10	In patients with moderate-to-severe and active GO, treatment of GO should be priority, and euthyroidism should be promptly restored and stably maintained	1, ØØØO
11	In patients with moderate-to-severe and active GO, large, multicenter randomized clinical trials should be designed to establish whether the conservative or the ablative approach is preferable for the long-term outcome of GO	1, ØOOO
12	In patients with moderate-to-severe and inactive GO, treatment of hyperthyroidism should be independent of residual GO manifestations	1, ØØOO
13	Hyperthyroid patients with sight-threatening GO should be treated with antithyroid drugs until dysthyroid optic neuropathy or corneal breakdown is cured and GO is inactive	1, ØØOO

## Methods

### Literature search

The major source of data acquisition included PubMed search strategies. Papers published in the last 35 years were screened. In addition, the bibliographies of relevant citations and chapters of major textbooks were evaluated for any additional appropriate citation.

### Grading

The GRADE system was used to make recommendations and express the quality of the evidence [9]. The task force used the following coding system: (1) indicates a strong recommendation and is associated with the sentence “we recommend”; (2) denotes a weak recommendation and is associated with the sentence “we suggest”. Evidence grading: ØOOO denotes very low quality evidence; ØØOO, low quality; ØØØO, moderate quality; ØØØØ, high quality.

## Effects of different modalities of treatment for hyperthyroidism on GO

### Antithyroid drugs

ATDs (thionamides: methimazole, carbimazole, propylthiouracil) are the first-line treatment for Graves’ hyperthyroidism in Europe [10] and Japan [11], while North Americans still prefer RAI [11], although the use of ATDs is lately increasing in USA as well [12]. ATDs usually bear a low rate of adverse events, but their major drawback is the high frequency of disease recurrences [13–15]. ATDs per se do not appear to influence the natural course of GO once euthyroidism has been restored. In a randomized control trial comparing RAI and ATDs, most patients had stable GO during ATD treatment [16], with a few cases of progression or remission compatible the natural history of the disease. This was confirmed by a recent observational, prospective study of a large series of newly diagnosed Graves’ patients undergoing an 18-month course of ATDs [3]. ATDs might, however, beneficially affect GO only as a consequence of the restoration of euthyroidism [5] and the associated progressive reduction in TSH-receptor antibody (TRAb) concentrations [17]. Fluctuations of thyroid status in fact may negatively affect GO. Therefore, assessment of thyroid status should be frequent (every 6–8 weeks) during the initial phases of treatment (or after changes in daily dose of the ATD) and periodical (every 3–4 months) thereafter. Hypothyroidism can also cause progression of GO [6]. There is no evidence that the choice of the regimen of ATD treatment (titration method vs. block-and-replace method) makes any difference in terms of GO course.

*Recommendation 1* We recommend that in patients with newly diagnosed Graves’ hyperthyroidism, euthyroidism be promptly restored by ATDs, and then stably maintained (1, ØØØØ);

*Recommendation 2* We recommend that thyroid status be assessed frequently during the initial phase of ATD treatment and regularly thereafter, to avoid fluctuations in thyroid status potentially detrimental for GO (1, ØØØO).

### Radioiodine

Radioiodine is an effective, widely used, and safe modality of treatment for Graves’ hyperthyroidism, employed as first-line therapy in North America [1, 11]. Hypothyroidism develops in the large majority of patients within 1 year from RAI administration [18].

The effects of RAI on GO are debated, due to the limited number of controlled studies [19]. In a small randomized clinical trial, not including a control group of patients on ATD treatment, post-RAI worsening of GO was observed in about one-third of patients not receiving concomitant oral prednisone treatment (see below), but in none of those treated with prednisone [20]. Subsequently, in another randomized clinical trial, GO progressed more frequently after RAI (33 %) than after thyroidectomy (16 %) or ATDs (10 %) [21]. In a large randomized clinical trial on 450 Graves’ patients, progression of GO was confirmed in about 15 % of patients after RAI, often transiently and most frequently in active smokers, but not after ATDs [16]. A more recent, large randomized clinical trial showed that both RAI and smoking are relevant risk factors for progression and also de novo development of GO [22], and smokers receiving RAI treatment have the highest risk [22]. Severity of hyperthyroidism [21], late correction of post-RAI hypothyroidism [23, 24], and probably, but not certainly, high TRAb concentrations [25–27] and its rise after RAI therapy [17] may also be relevant risk cofactors. The absence of GO prior to RAI administration does not rule out the possibility of its occurrence after RAI treatment [22], but the risk of progression is higher in patients with preexisting GO [16]. Recent onset of hyperthyroidism may represent an additional risk factor and should be taken into account, particularly if patients are given RAI as first-line treatment [22, 27].

In patients at risk of RAI-associated GO occurrence or progression, oral steroid prophylaxis is almost universally effective [28]. This was shown by two randomized clinical trials [16, 22] and confirmed by two meta-analyses [29, 30]. Steroid prophylaxis can be carried out using very low doses of prednisone (0.2 mg/kg bodyweight), given 1 day after RAI therapy, gradually tapered down and withdrawn after 6 weeks [31]. As indicated by the results of a recent meta-analysis of 8 trials including 850 patients, steroid prophylaxis probably can be avoided in patients with absent or inactive GO [30]. This is particularly true if other risk factors for RAI-associated progression of GO are absent [7]. As mentioned above, short duration of Graves’ hyperthyroidism, might represent an important case in favor of steroid prophylaxis also in these patients [27]. Because GO may newly occur after RAI treatment, it is always wise to discuss the pros and cons of steroid prophylaxis also in this category of patients [32].

*Recommendation 3* We recommend steroid prophylaxis in patients receiving RAI treatment, if mild and active GO preexists or there are risk factors for RAI-associated GO development or progression (1, ØØØØ);

*Recommendation 4* We recommend that pros and cons of steroid prophylaxis after RAI treatment be thoroughly discussed also with patients with absent or inactive GO prior to RAI treatment (1, ØOOO);

### Thyroidectomy

Thyroidectomy is an effective, but less commonly used modality of treatment for Graves’ hyperthyroidism [1, 11]. As shown by three meta-analyses, near-total or total thyroidectomy is associated with a lower incidence of relapsing hyperthyroidism [33–35], with no [33] or minor [34, 35] differences in the rate of complications (hypoparathyroidism, laryngeal nerve palsy). Accordingly, near-total/total thyroidectomy, performed by a skilled surgeon, should be regarded as the procedure of choice for Graves’ patients. A recent systematic review of existing literature suggested that surgery is more successful than RAI, as the definitive treatment for Graves’ hyperthyroidism [36].

As to the effects of thyroid surgery on GO, a randomized clinical trial [21] showed that the rate of de novo occurrence or progression of GO among patients submitted to subtotal thyroidectomy or ATD treatment was similar, but significantly lower than that observed after RAI treatment. In a case–control prospective study, near-total thyroidectomy did not cause significant variations in ocular involvement in 17 of 18 patients, possibly due to short-term release of thyroid antigens and immediate removal of autoreactive T lymphocytes [37]. A prospective study of 48 Graves’ patients treated by total thyroidectomy showed that GO improved after surgery in 90 % of patients with preexisting GO [38]. A recent meta-analysis of randomized clinical trials failed to show any significant difference between total thyroidectomy and subtotal thyroidectomy with regard to the course of GO [35].

*Recommendation 5* We recommend that, if surgery is selected, near-total/thyroid thyroidectomy should be preferred to subtotal thyroidectomy, because the former is associated with a higher rate of successful treatment of hyperthyroidism and with no differences in the outcome of GO; steroid prophylaxis is not required (1, ØØØØ).

### Total thyroid ablation

Surgery and RAI treatment can be used sequentially to achieve total thyroid ablation (TTA), as in patients with differentiated thyroid cancer. The latter might be beneficial for GO through complete removal of autoreactive T lymphocytes and thyroid antigen(s) shared by the thyroid gland and the orbital tissue probably involved in the pathogenesis of GO [39]. A randomized clinical trial of 60 patients with mild to moderate-severe and active GO were treated with near-total thyroidectomy alone or TTA, and concomitantly received intravenous glucocorticoids combined with orbital radiotherapy for GO [40]. TTA was associated with a short-term better GO outcome (particularly on lid aperture and exophthalmos) [40]. However, a follow-study on the same series failed to show significant differences in the long term [41]. A recent prospective, randomized, single-blind clinical trial on 40 patients (treated with intravenous glucocorticoids for moderate-to-severe GO) showed that TTA was more effective than surgery alone in achieving an earlier and steady improvement of GO [42]. Neither study [40, 42] was performed in the absence of a concomitant treatment for GO. Two retrospective studies also suggested beneficial effects of TTA on GO [43, 44]. Evidence on the effects of early TTA in patients with no or mild GO is lacking.

*Recommendation 6* We suggest that, if surgery for Graves’ hyperthyroidism is selected in patients with GO, post-operative remnant ablation be considered, because this inactivates the disease earlier and allows prompter rehabilitative surgery, if needed (2, ØØØO).

## Choice of thyroid treatment in patients with GO

The natural history of GO is characterized by an early inflammatory phase (active GO), a plateau phase, and a spontaneous (although incomplete) remission (inactive GO), the whole cycle likely lasting 18–24 months [2]. Assessment of activity relies on a useful, although imperfect tool, the Clinical Activity Score (CAS), which includes 7 items (eyelid edema, eyelid erythema, conjunctival redness, chemosis, edema of the caruncle, spontaneous ocular pain, pain with ocular movements): GO is considered active if at least three out of seven items are present (CAS ≥ 3/7) [7]. Assessment of severity is based on a global evaluation of soft tissue changes, exophthalmos, ocular dysmotility, optic nerve involvement, and corneal breakdown [7]. Accordingly, GO can be active or inactive, mild, moderate-to-severe, or sight-threatening [7].

### Mild and active GO

A wait-and-see strategy is sufficient in most cases, although occasional patients may need immunosuppressive treatment [7], because of a negative impact on their quality of life [45, 46]. A recent randomized clinical trial of patients treated with ATDs showed that a 6-month selenium supplementation helps to improve mild GO and to prevent its progression to more severe forms, and is devoid of any relevant side effect [47]. Selenium supplementation might be useful also in patients who have mild and active GO and are treated with RAI or thyroidectomy, but this remains to be demonstrated [48]. Treatment of hyperthyroidism in these patients is independent of GO and relies on established criteria (age, goiter size, first episode of hyperthyroidism *vs*. relapse, patient’s preference, etc.) [14] or regional differences [1, 11]. For the time being, there is no evidence from randomized clinical trials that the long-term outcome of GO is better using ATDs or thyroid ablation. Steroid prophylaxis is indicated in selected cases only if RAI treatment is selected [30].

*Recommendation 7* We recommend that patients who have mild and active GO and are treated with ATDs receive a 6-month selenium supplementation (1, ØØØO).

*Recommendation 8* We recommend that the modality of treatment for hyperthyroidism in patients with mild and active GO be selected independently of GO (1, ØOOO).

### Mild and inactive GO

In these patients rehabilitative surgery for cosmetic or functional reasons (orbital decompression, squint surgery, eyelid surgery) may be needed. Treatment for hyperthyroidism is unlikely to cause ocular changes and, therefore, is chosen independently of GO [8]. If RAI treatment is selected, steroid prophylaxis is not indicated unless risk factors for RAI-associated GO progression exist [30].

*Recommendation 9* We recommend that the modality of treatment for hyperthyroidism in patients with mild and inactive GO be selected independently of GO (1, ØOOO).

### Moderate-to-severe and active GO

These patients should receive prompt therapies for GO, because treatment outcome is inversely correlated to disease duration [2]. Glucocorticoids, preferably administered through the intravenous route [49, 50], represent the first-line treatment, with or without associated orbital radiotherapy [51, 52]. Novel treatments are under evaluation. Among them, rituximab [53]: two recent randomized clinical trials have provided conflicting results, no effect [54] vs beneficial effect [55], indicates the need for larger multicenter studies. The choice of the optimal thyroid treatment in these patients is a matter of debate. Although not supported by randomized clinical trials, an important argument in favor of ATDs is that prompt correction of hyperthyroidism and stable maintenance of euthyroidism, usually achieved with ATDs, are per se beneficial for GO [5]. Accordingly, one line of thinking is that treatment of GO should be the priority, while patients are long-term treated with ATDs and definitive treatment for hyperthyroidism, if needed, is postponed after inactivation and cure of GO [56, 57]. On the other hand, ATD treatment is associated with a high relapse rate after drug withdrawal [1], and the continuing thyroid activity and fluctuations in thyroid status might negatively influence the course of GO [5, 39]. Thus, a second line of thinking suggests that, after control of hyperthyroidism with ATDs, even in these patients the thyroid should be ablated while GO is concomitantly managed with immunosuppression [40–44]. Even if two randomized clinical trials suggest a short-term advantage of this approach, long-term results are not fully convincing. For the time being, evidence is lacking as to the superiority of the conservative approach over the ablative approach and vice versa. Accordingly, appropriately powered randomized clinical trials should be performed to address this important issue.

*Recommendation 10* We recommend that in patients with moderate-to-severe and active GO, treatment of GO should be priority, and euthyroidism should be promptly restored and stably maintained (1, ØØØO).

*Recommendation 11* We recommend that in patients with moderate-to-severe and active GO, large, multicenter randomized clinical trials be designed to establish whether the conservative or the ablative approach is preferable for the long-term outcome of GO (1, ØOOO).

### Moderate-to-severe and inactive GO

In these patients the choice of thyroid treatment is on standard criteria largely independent of GO. If RAI is selected, steroid prophylaxis can be avoided if other risk factors for RAI-associated GO progression, particularly smoking, are absent [30]. A strict post-RAI follow-up is needed to avoid periods of persisting hyperthyroidism or uncontrolled hypothyroidism [23, 24].

*Recommendation 12* We recommend that in patients with moderate-to-severe and inactive GO, treatment of hyperthyroidism is independent of residual GO manifestations (1, ØØOO).

### Sight-threatening GO

This is a real endocrine emergency because patients are at risk of sight loss due to dysthyroid optic neuropathy (DON) and/or corneal breakdown. Therefore, they should immediately be treated with high-dose intravenous glucocorticoids and subsequent orbital decompression if response to steroids is poor or absent within 2–4 weeks [58, 59] or if there are signs of persistent inflammatory activity or optic disc swelling. Hyperthyroidism must be treated with ATDs and definitive treatment with RAI or thyroidectomy, if needed, postponed until DON and/or corneal breakdown has been cured and GO is inactive [7].

*Recommendation 13* We recommend that hyperthyroid patients with sight-threatening GO be treated with ATDs until DON or corneal breakdown is cured and GO is inactive (1, ØØOO).

## Conclusions

Optimal treatment of hyperthyroidism due to Graves’ disease in patients with associated GO remains an unsolved dilemma in many instances, because of the scarcity of randomized clinical trials. When GO is mild (either active or inactive) or when GO is moderate-to-severe, but stably inactive, treatment of hyperthyroidism is largely independent of GO and based on established criteria. If GO is sight-threatening, its treatment should be immediate, and hyperthyroidism should be controlled with ATDs. The major field of controversy is represented by moderate-to-severe and active GO, because GO should be promptly treated, but it is unclear whether management of hyperthyroidism should preferably be conservative or ablative.
